# Quantum dot assisted luminescent hexarhenium cluster dye for a transparent luminescent solar concentrator

**DOI:** 10.1038/s41598-021-93223-7

**Published:** 2021-07-05

**Authors:** Jun Choi, Kyungkon Kim, Sung-Jin Kim

**Affiliations:** Department of Chemistry and Nano Science, Ewha Womans University, Seoul, 120-750 South Korea

**Keywords:** Energy, Photochemistry, Nanoscale materials

## Abstract

A luminescent solar concentrator (LSC) is a solar-light harvesting device that concentrates light on a photovoltaic cell placed at the edge of an LSC panel to convert it into electricity. The nano-sized inorganic–organic cluster complex (dMDAEMA)_4_[Re_6_S_8_(NCS)_6_] (this refers to RMC where dMDAEMA is 2-dimethyl amino ethyl methacrylate) is a promising candidate for LSC luminophores due to its downshifted broad photoluminescence suitable for photovoltaic cells. However, the low quantum yield (QY) of RMC limits the performance. Here, zinc-doped CuGaS/ZnS core/shell quantum dots (ZQD) were used as energy transferring donor with high QY to improve the performance of the LSC. The two metal chalcogenide luminophores, RMC and ZQD, are chemically suitable for dispersion in an amphiphilic polymer matrix, producing a transparent waveguide with suppressed reabsorption and extended harvesting coverage of the solar spectrum. We achieved an *η*_*opt*_ of 3.47% and a PCE of 1.23% while maintaining greater than 80% transparency in the visible range. The high performance of this dual-dye LSC with suppressed reabsorption, and scattering losses is not only due to uniform dispersion of dyes in a polymer matrix, but also energy transfer from ZQD to RMC. This report suggests a new possibility for promising various multi-dye LSCs for use in building-integrated photovoltaic windows.

## Introduction

**Figure 1:**
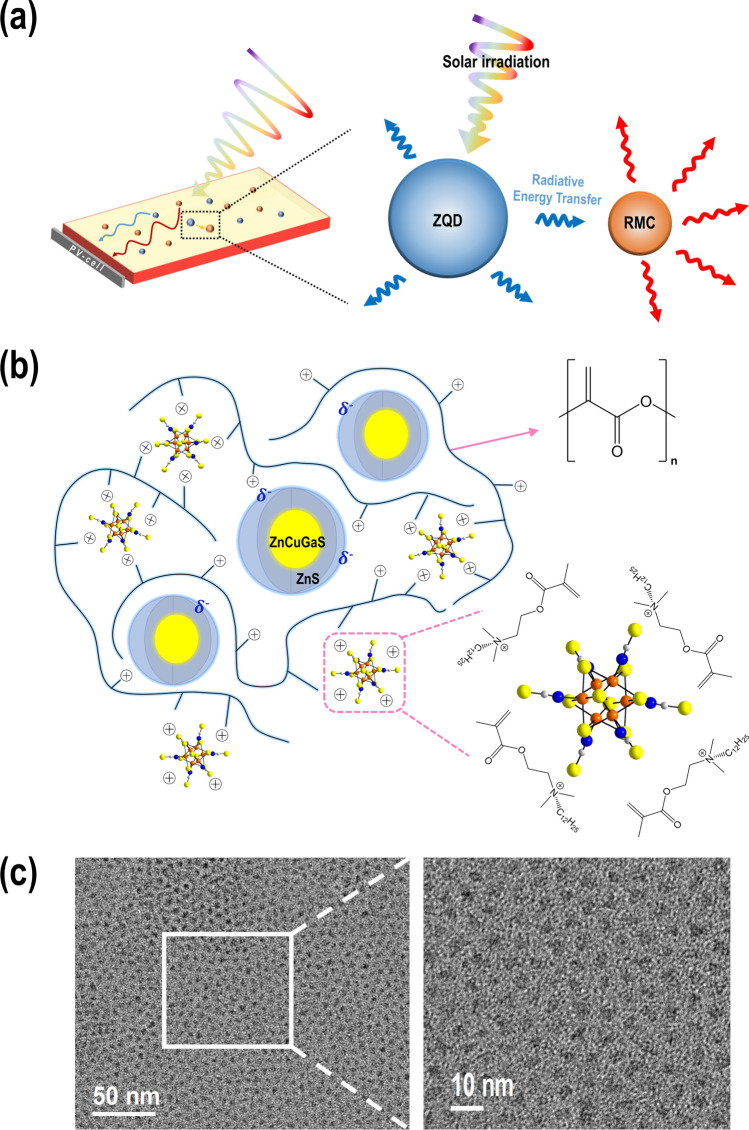
(**a**) Schematic representation of the dual-luminophore LSC system showing energy transfer between luminophores. (**b**) Schematic representation of RMC- and ZQD-embedded polymer waveguide. Organic dMDAEMA^+^ cations were polymerized such that the metal cluster dyes were anchored on the polymer chain without cluster–cluster contact in the PMMA matrix. (**c**) TEM image of ZQD core/shell quantum dots.

Clean-energy technologies are becoming increasingly important due to concerns over global warming and depletion of fossil-fuel resources. Silicon-based photovoltaic (PV) technologies are among the most widely used options for generation of renewable electricity^1^. However, conventional silicon-based photovoltaic technology suffers from poor transparency, high weight, and the need for large areas to accommodate the PV arrays. Luminescent solar concentrator (LSC) technology first appeared in 1976, but it has received renewed attention in recent years as an effective complement to conventional silicon PV systems due to its multiple advantages, including color options, transparency, and ease of installation on facades of urban buildings^2–5^. The incidence angle of solar light is less limited in LSCs compared with silicon PV cells, which means fewer restrictions on where and how to install such systems. An LSC consists of a polymer waveguide slab containing luminophores with a refractive index of approximately 1.5 and PV cells attached at the side edges of the waveguide^6,7^. This device absorbs incident sunlight on the front surface of the waveguide and uses internal reflection to concentrate re-emitted light at longer wavelengths onto a small area of PV cells at the edges of the slab. The emitted light generates electricity as depicted in Fig. 1a.

Despite considerable researches into applying various organic luminescent dyes to LSCs to ensure high quantum efficiency, practical application in large-area devices such as PV windows has been limited by self-absorption losses and strong colors in the visible range. For example, a typical organic dye such as Lumogen 305 used in state-of-art LSCs has a relatively small downshift of 140 meV, resulting in high reabsorption loss. In addition, organic dyes have low stability under ultraviolet (UV) light, hindering creation of LSCs with long-term stable power conversion efficiency (PCE)^8–10^.

Inorganic quantum dots are stable under UV light, and their high quantum efficiencies are suitable for high-PCE applications. However, it has proven difficult to uniformly disperse inorganic luminescent quantum dots (QDs) in a non-polar polymer matrix. LSC devices with QDs such as PbS/CdS^11^ and CdSe/CdS^7,12–16^ dispersed in LSC polymer waveguides have achieved optical efficiencies of 6.1% and 1.9%, respectively. However, QDs with heavy metals such as cadmium and lead have strong color and toxicity concerns. Recently, studies have focused on heavy metal–free QDs^17^ such as CuInS_2_/ZnS^18,19^, CuInSe_1−x_S_x_^20^, carbon dots^13^, and silicon nanocrystals (SiNC)^21–25^. As inorganic QDs for LSCs, perovskite materials have also been reported^26,27^. Perovskites are characterized by high QYs, and negligible self-absorption resulting in higher optical performance. However the problems for LSC application are long term stability in humid environments and toxicity issues. In this study, we employed non-toxic zinc-doped CuGaS/ZnS core/shell QDs, which have a high quantum yield (QY) of up to 80% and emissions that are tunable according to doping amount of zinc in CuGaS cores with ZnS shells.

As molecular QDs, M_6_X_8_L_6_-type (M = Mo, W, Re, X = S, Se, L = halogen) hexanuclear metal clusters with a massive downshift and weak absorption in the visible range have been reported as luminophores^28–30^. However, LSCs with these luminophores show low efficiency because of their low QY. For example, the PCE of an LSC using nano-sized hexamolybdenum metal halide clusters such as K_2_Mo_6_Cl_14_ and (TBA)_2_Mo_6_Cl_14_ has reached 0.44%^31^^,^^32^.

It has been reported that multi-dyes with different absorption ranges in visible light can be used to improve optical efficiency of LSCs by extending the emission range^33–35^. However, the previously reported multi-dye LSCs are mostly consisted of organic dyes, which are disadvantageous in terms of colorless high transparency and reabsorption loss. Furthermore, since organic dyes have poor stability to UV light, it is difficult to apply them in practical use.

Here, we present an LSC with two chemically comparable, optically synergetic, and photo stable metal chalcogenide dyes: the hexarhenium cluster organic–inorganic salt (dMDAEMA)_4_[Re_6_S_8_(NCS)_6_] (RMC) and zinc-doped CuGaS/ZnS core/shell quantum dots (ZQD). Both RMCs and ZQDs are chemically polar, commonly absorb light in the UV-A region of the solar spectrum (λ = 350–400 nm), and emit strong and broad photoluminescence between 400 and 900 nm. In our LSCs the two dyes were uniformly dispersed in an amphiphilic polymer matrix by electrostatic and polar interactions. Solar energy strongly emitted by ZQDs could extend the energy harvesting range and also could be transferred to RMCs, enhancing the efficiency of the LSC. The synergetic performance of a dual dye–containing LSC was compared with single-dye LSC systems using either RMCs or ZQDs.

## Results and discussion

### Synthesis of LSC dyes

To produce highly transparent LSCs, luminophores must be uniformly dispersed without aggregation in the polymer waveguide. We prepared an amphiphilic inorganic–organic salt RMC consisting of the nano-sized inorganic cluster anion [Re_6_S_8_(NCS)_6_]^4−^ and the polymerizable organic cation [2-(methacryloyloxy)-ethyl]-dimethyl-dodecylammonium, (dMDAEMA)^+^. This salt was copolymerized with the organic solvent methyl methacrylate (MMA) to become an inorganic–organic hybrid of RMC and poly methyl methacrylate (PMMA). The methacrylate functional group at one end of the organic cation dMDAEMA^+^ is suitable for copolymerization with MMA to produce an amphiphilic polymer matrix. The other end of the cation has a long aliphatic C_12_H_15_ chain that improves solubility in MMA. To compensate for the shortcomings associated with the low QY of the RMC, ZQDs with a high QY were embedded within the polymer matrix. As a single dye, ZQD concentration increased within the PMMA, and scattering of emitted light increased as a result of agglomeration of ZQDs. However, when two dyes were dispersed together, such scattering was reduced. In a dual dye–embedded LSC waveguide, [Re_6_S_8_(NCS)_6_]^4−^ anionic metal clusters were immobilized on the cationic (dMDAEMA)^+^ sites of the polymeric backbone, and also ZQDs were well dispersed around the cationic sites of the polymer due to their polar sulfide surfaces. Isolation of each dye without agglomeration within the host matrix is important to preserve two intrinsic optical characteristics without non-radiative self-quenching. (Fig. 1b). Because RMC-encapsulated polymer plates strongly absorb in the UV-A spectral region (near 400 nm), they do not have a strong color and exhibit broad emissions at 500–900 nm. The ZQDs also absorb in the UV-A region, show strong emissions at 400–700 nm, and have a high QY, indicating their promise as materials for LSC dyes. We prepared ZQDs with ZnS shells on zinc-doped CuGaS_2_ cores to optimize LSC performance by synergetic energy transfer to the cluster dye and to cover a broader harvesting range for silicon PV cells. The optimized ZQDs achieved a QY of approximately 78%, which is superior to those other similar QDs. Also, they exhibited a large Stokes shift minimizing a source of reabsorption. Transmission electron microscopy (TEM) images of ZQDs revealed spherical shapes with an average size of approximately 3.62 nm and uniform size distribution (Fig. 1c, Figure S1).

### Characterization of dyes

**Figure 2:**
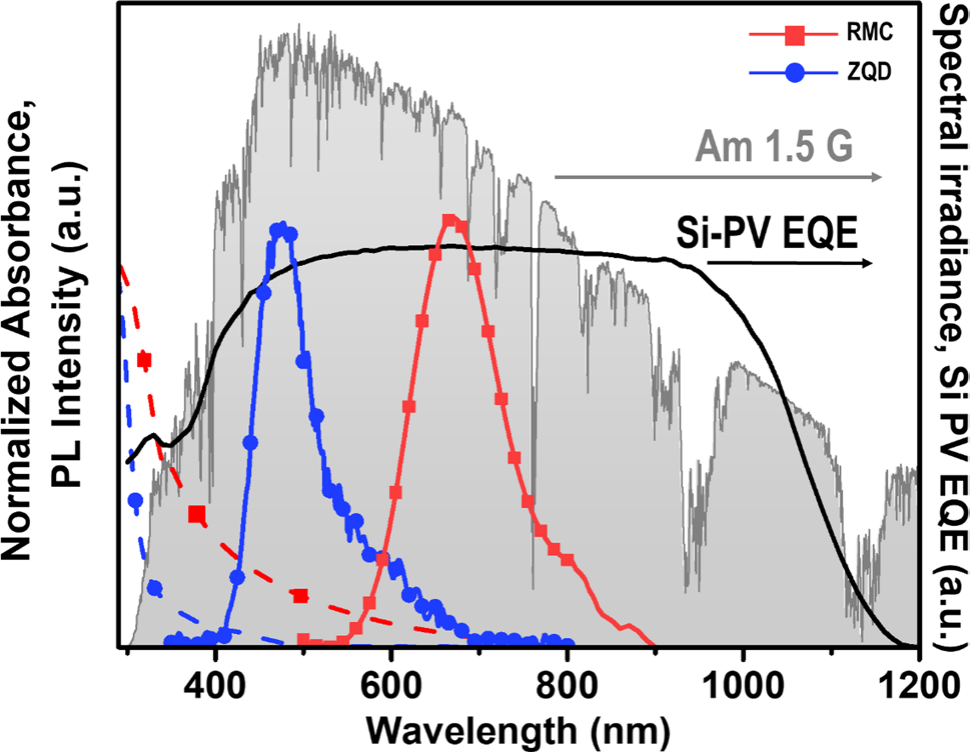
Normalized absorption (dashed line) and PL (solid line) spectra of RMC (red square) and ZQD (blue circle) dissolved in an argon-saturated tetrahydrofuran solution. AM 1.5 G solar spectrum (gray shading) and a silicon-PV EQE spectrum (black solid line) are shown.

Normalized absorption and emission spectra of RMC and ZQD in tetrahydrofuran (THF) solution are shown in Fig. 2. The maximum emission peak of RMC at 670 nm and that of ZQD at 471 nm were comparable to the absorption range of a silicon PV cell. RMC has high absorption in the UV range, a largely downshifted phosphorescence, and long excited state lifetimes^36^. Such a large downshift of photoluminescence (PL) by RMC can reduce reabsorption loss, which is a primary obstacle to practical application of large-scale LSC devices. RMC and ZQD have high extinction coefficients at 345 nm of 8.41 × 10^4^ and 7.72 × 10^3^ M^−1^ cm^−1^, respectively, which are comparable to those of semiconducting PV materials such as GaAs and silicon. The absorption and emission spectra of the RMC and ZQD mixture in the solid hybrid of LSC plate are shown in Figure S2, in which the PL peak of ZQD is red-shifted to 490 nm compared with that of ZQD alone at 471 nm, indicating interaction between the two dyes.

Because RMC exhibits significant absorption between 400 and 600 nm, it can absorb the strongly emitted energy from ZQD at 471 nm as an energy acceptor. To investigate the contribution of ZQD as an energy donor to the efficiency of dual-dye LSC devices, we added 0.08 mg/mL of ZQD solution to RMC solutions at various concentrations from 0 to 1.13 mg/mL. Tetrahydrofuran was selected as a solvent to disperse both polar ZQD and RMC dyes. The absorption spectra between 280 and 800 nm and emission spectra of 350 to 900 nm are shown in Figure S3a and b. As the concentration of RMC increased at a constant ZQD concentration, the emission intensity of ZQD at 490 nm decreased and emissions of RMC at 670 nm increased, indicating that part of the energy emitted by ZQD was absorbed by RMC^9,37^. Later, we found that LSC fabrication with 0.08 mg/mL of ZQD and 0.5 mg/mL of RMC achieved the highest performance of LSC device. The PL quenching efficiency of donor and energy sensitization efficiency of acceptor were estimated from the PL intensities of ZQD and RMC. We estimated the efficiency of energy transfer between two luminophores and summarized in Table S1. The highest efficiency of energy transfer 22% was obtained from sample number 5 with concentration ratio of ZQD:RMC = 0.08:0.50^38^. Donor quenching increased according to acceptor concentration; however, acceptor sensitization reached a maximum at a ZQD:RMC concentration ratio of 0.08:0.50 (mg/mL). This indicates that non-radiative energy transfer may occur at concentrations of RMC higher than 0.5 mg/mL. The optimal concentration ratio of ZQD:RMC for efficient energy transfer and sensitization was found to be 0.08:0.50 (mg/mL) (Figure S3c).

### LSC preparation and optical properties

**Figure 3:**
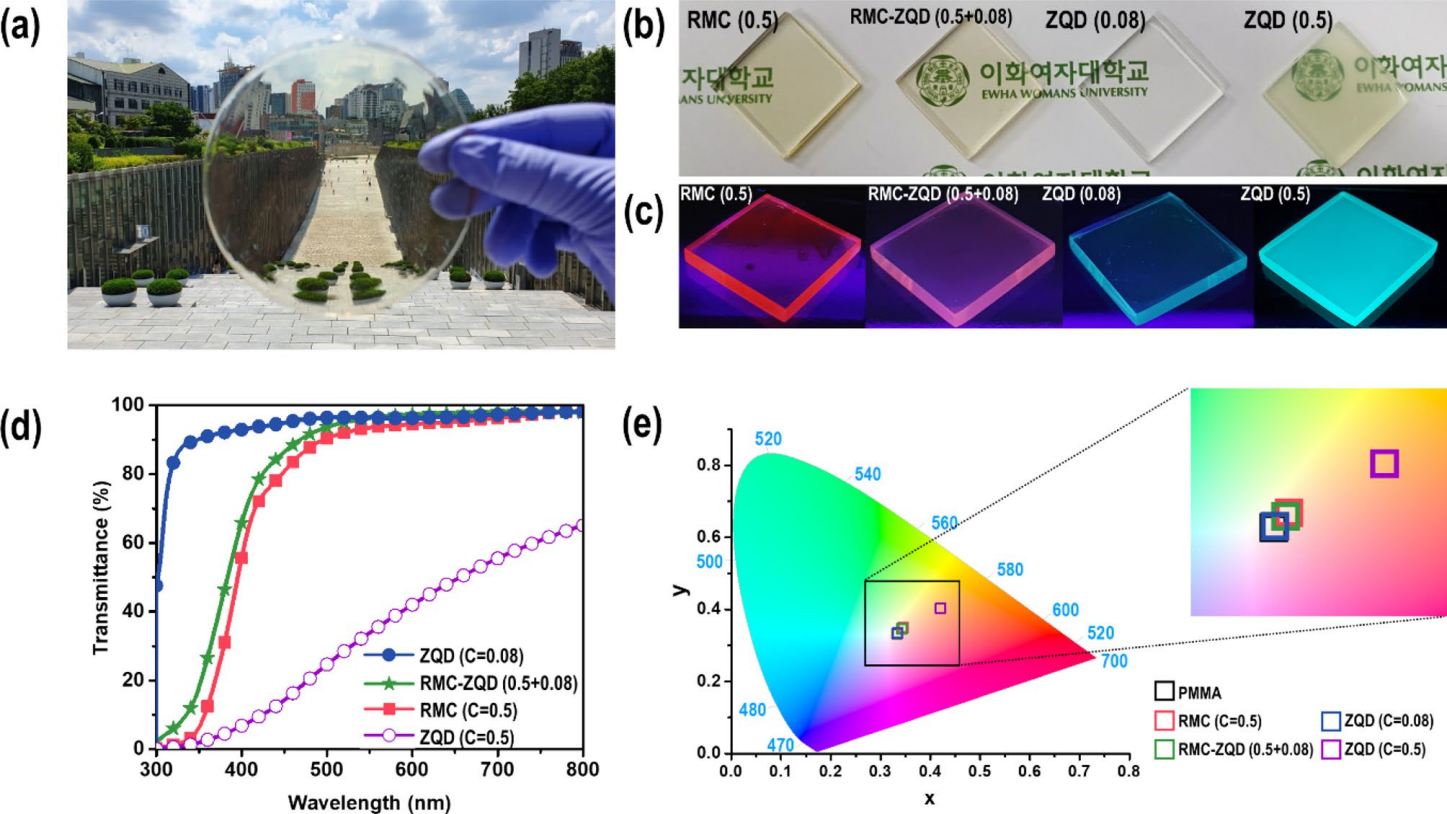
(**a**) Image of the 12-cm-diameter LSC fabricated with dual dyes (RMC and ZQD). (**b**) Photos of the LSC with RMC and ZQD as single luminophores and LSC containing dual dyes in daylight. From the left, the LSC contains 0.5 mg/mL of RMC, 0.5 mg/mL of RMC and 0.08 mg/mL of ZQD, 0.08 mg/mL of ZQD, and 0.5 mg/mL of ZQD. All LSC plates measure 2.5 cm × 2.5 cm × 0.3 cm^3^. (**c**) Photos of the same LSCs under UV light (365 nm). (**d**) UV–vis transmittance spectra of the same LSCs. (**e**) A CIE 1931 color space chromaticity diagram indicating the color coordinates of the LSCs.

We fabricated dual-dye LSCs with a mixture of RMC and ZQD. For comparison purposes, we also fabricated two single-luminophore-encapsulated LSCs with various concentrations of RMC and ZQD. LSCs with RMC were transparent at high concentrations, although a ZQD-encapsulated LSC showed transmittance as high as that of the RMC until the concentration of ZQD was lower than 0.33 mg/mL (Figure S4). LSC plates with a ZQD concentration greater than 0.33 mg/mL were hazy and showed reduced transmittance due to scattering of ZQD aggregates in a non-polar PMMA matrix. The aggregation of dyes is a primary factor in scattering solar light, and it can be difficult to efficiently concentrate light on PV cells as LSC plate size increases. A high concentration of quantum dots, 0.5 mg/mL of ZQD, significantly reduced transparency of LSCs. Comparing with ZQD alone LSC, the LSC encapsulated with two dyes achieved high transmittance in the visible range without haziness, as shown in Fig. 3a,b indicating no aggregation of dyes. An LSC containing a very low concentration of 0.08 mg/mL of ZQD was colorless and exhibited almost the same transparency as the blank PMMA used as a reference. LSCs under UV illumination (365 nm) are shown in Fig. 3c. When irradiated with UV light, strong PL concentrated at the edge of the LSC waveguide, with the exception of the embedded 0.5 mg/mL of ZQD. The LSC containing 0.5 mg/mL of ZQD scattered PL in all directions. As seen in Fig. 3d, all samples except ZQD of 0.5 mg/mL maintained high transparency in the visible-light region. We also calculated color coordinates and analyzed the color rendering index (CRI) as defined by the International Commission on Illumination 1931 chromaticity diagram. The color coordinates of all LSCs (Table S2) were located near the central region (0.3, 0.3) of the chromaticity diagram, as shown in Fig. 3e. All samples exhibited a nearly neutral color sensation, with only a small shift toward the yellow region. In particular, the RMC-ZQD dual-dye LSC plates exhibited CRI values similar to those of the blank PMMA without any luminophores. The CRI values ranged from 99% for the blank PMMA to 97% for the LSC sample containing dual dyes. When CRI values exceeded 80%, the plates were considered suitable for application to building-integrated photovoltaic windows, and all LSC plates prepared in this experiment had relatively high CRI values higher than 90%^21^.

### Luminescent solar concentrator efficiency

**Figure 4:**
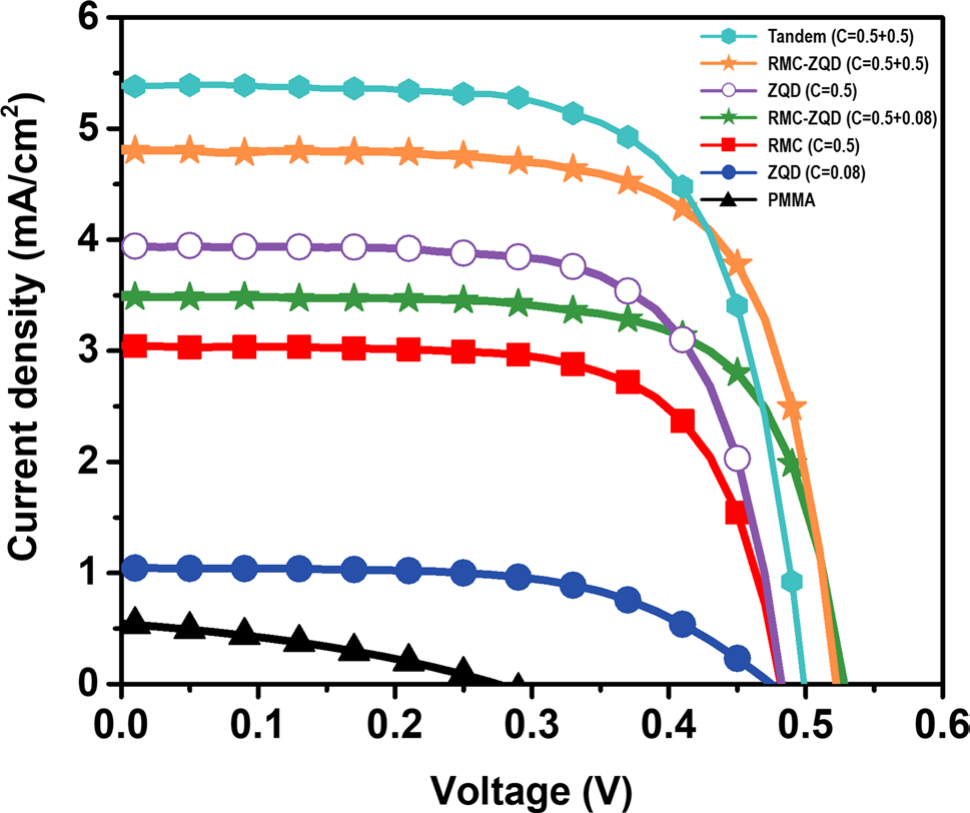
Current–density voltage (J–V) curves of LSC devices with various concentrations of luminophores: C = 0.5 mg/mL of RMC (red line), C = 0.5 mg/mL of ZQD (purple line), C = 0.08 mg/mL of ZQD (blue line), RMC-ZQD with 0.5 mg/mL RMC and 0.08 mg/mL ZQD (green line), RMC-ZQD with 0.5 mg/mL RMC and 0.5 mg/mL ZQD (yellow line), tandem structure RMC + ZQD with 0.5 mg/mL RMC and 0.5 mg/mL ZQD (cyan line), and C = 0.0 mg/mL PMMA (black line).

The performance of LSC-system can be described by power conversion efficiency (PCE), which is defined as the ratio of the electrical power output to the incident power (P_in_). For PCE measurements, LSC plates with dimension of 2.5 cm × 2.5 cm × 0.3 cm were prepared. Illumination with an intensity of 1 sun to simulate sunlight was vertically irradiated on a 2.5 cm × 2.5 cm front surface of an LSC, and current voltage characteristics of a silicon PV cell at the edges (2.5 cm × 0.3) were investigated. These silicon PV cells were mounted at the four edges of LSC plates in parallel. The LSCs with one luminophore (RMC or ZQD) were fabricated and evaluated as a function of concentrations, and plotted in Figure S5 and S6. For comparisons, some of representative single and dual-dye LSCs’ J–V curves and PV parameters including current density (*J*_*SC*_), open-circuit voltage (*V*_*OC*_), and fill factor (*FF*) of edge-attached PV cells are summarized in Fig. 4 and Table 1. The PCE was calculated by *J*_*SC*_ × *V*_*OC*_ × *FF*/*P*_*in*_. Values for *J*_*SC*_ were obtained by dividing short-circuit current (*I*_*SC*_) by the front illuminated area of the LSC^8,31,39–41^. In the LSC with only RMC, the highest PCE obtained was 0.96 ± 0.06% at an RMC concentration of 0.5 mg/mL. The J_SC_, the amount of electric current flowing per unit cross-sectional area of the LSC, was 3.04 ± 0.01 mA cm^−2^. For the LSC with ZQD, the PCE values were 0.29 ± 0.05% at a concentration of 0.08 mg/mL and 1.24 ± 0.05% at 0.5 mg/mL. The J_SC_ values were 1.03 ± 0.03 mA cm^−2^ and 3.96 ± 0.02 mA cm^−2^ at 0.08 and 0.5 mg/mL, respectively.

**Table 1:** Photovoltaic parameters obtained from PCE measurements. The short-circuit current density (*J*_*SC*_) was estimated based on LSC front surface areas of 2.5 cm × 2.5 cm, and 4 PV cells were attached in parallel.

	J_SC_ (mA cm^−2^)	V_OC_ (V)	FF	PCE (%)	$\eta _{{{\text{OPT}}}}$ (%)
RMC-ZQD (C = 0.5 + 0.5)	4.805	0.504	0.697	1.69	4.77
RMC-ZQD (C = 0.5 + 0.08)	3.486	0.508	0.694	1.23	3.46
ZQD (C = 0.5)	3.961	0.459	0.683	1.24	3.93
RMC (C = 0.5)	3.040	0.463	0.681	0.96	3.02
ZQD (C = 0.08)	1.025	0.431	0.651	0.29	1.02
PMMA	0.54	0.277	0.343	0.05	0.71
Tandem ZQD(C = 0.5) + RMC (C = 0.5)	5.404	0.499	0.689	1.85	5.36

The PCE and *J*_*SC*_ of the dual-dye LSC of 0.08 mg/mL of ZQD and 0.5 mg/mL of RMC were higher than those of each single-dye LSC, 1.23 ± 0.05% and 3.49 ± 0.01 mA cm^−2^, respectively. The *V*_*OC*_ values in the dual-dye LSC were higher than those of single-dye LSCs, indicating a different optical band gap in the dual-dye hybrid system. Because *FF* is a characteristic parameter of solar cells as an important measure of quality, the increase in FF of dual-dye LSCs indicates improved quality of the device. The higher *V*_*OC*_ and *FF* values of the dual-dye LSC compared with those of single-dye systems indicate an optically synergetic effect between dyes. The LSC made of blank PMMA without any luminophores had a negligible PCE of 0.05 ± 0.02%, indicating that dyes primarily contribute to energy conversion in a PMMA matrix. The single-dye LSC with high concentration of 0.5 mg/mL of ZQD achieved a PCE similar to that of the dual-dye system consisting of 0.08 mg/mL of ZQD and 0.5 mg/mL of RMC. However, the ZQD-only LSC suffered significant haziness. The parameters of a dual-dye LSC with 0.5 mg/mL of ZQD and 0.5 mg/mL of RMC concentration also were measured for a comparison. The PCE of 1.69 ± 0.02%, and the J_SC_ of 4.81 ± 0.02 mA cm^−2^ were much higher than former dual-dye LSC. Two dual-dye systems with different ZQD concentrations were compared in terms of reabsorption loss in later section. For a comparison, we measured the photovoltaic parameters of a tandem structured LSC composed of two individually dye-encapsulated LSC sheets with each 3 mm thickness. The tandem structured LSC exhibited a higher current density resulting in higher PCE than corresponding dual-dye LSC. However, LSCs containing ZQD alone have optical disadvantages such as high scattering and reabsorption losses, so these important factors must be considered for practical applications.

The efficiency of an LSC can be evaluated in terms of optical efficiency (*η*_*opt*_). Optical efficiency is defined by applying energy-loss steps until the LSC receives solar energy and converts it into power conversion efficiency. The $\eta _{{OPT}}$ value can be defined as $\eta _{{OPT}}  = \smallint (1 - R(\lambda ))(1 - e^{{\alpha _{C} (\lambda )d}} )(1 - e^{{\alpha _{{wg}} (\lambda )L}} )\eta _{{trap}} (\lambda )\phi _{{PL}} (1 - \eta _{s} (\lambda ))(1 - \eta _{{RA}} (\lambda ))d\lambda$, where R($\lambda$) is the front slab reflection; α_C and_ α_wg_ are the absorption coefficients of the luminophore and matrix, respectively; *d* is the thickness of the slab; L is the length of the slab; $\eta _{{trap}}$ is the waveguide efficiency; $\phi _{{PL}}$ is the QY of the luminophore; $(1 - \eta _{s} (\lambda ))$ is the scattering events; and $(1 - \eta _{{RA}} (\lambda ))$ is the reabsorption process^2^. Optical efficiency can be expressed more simply in terms of the measured current of the LSC and PV cell.

1 $$ \eta _{{Opt}}  = \frac{{I_{{LSC}} }}{{I_{{SC}}  \times G}} $$

In Eq. (1), *I*_*LSC*_ and *I*_*SC*_ are the short-circuit current of the silicon PV cell coupled to the LSC and the short-circuit current of the same silicon PV cell under direct illumination of the same light source, respectively (Figures S7, S8)^11,13,40^. *G* is the geometric factor, which can be calculated by dividing the area of the front surface of the LSC by the area of the edge of the LSC to which the PV cell is attached. We fabricated LSCs with dimensions of 2.5 cm × 2.5 cm × 0.3 cm to estimate their $~\eta _{{OPT}}$ values. Each LSC was fully illuminated by a light source perpendicular to its surface using AM 1.5 G solar simulator (100 mW cm^−2^). The optical efficiencies of two dual-dye LSCs were 4.77% and 3.46% were higher than previously reported values of only inorganic QD LSCs i.e. 2.85% for Si QD^22^ and 3.27% for CuInSe_x_S_1−x_/ZnS^20^.

The QY measured from the ZQD-embedded LSC was approximately 78% at 345 nm, which was consistent with values presented previously^42^. The RMC-embedded LSC achieved a QY of 19% at an excitation wavelength of 345 nm. The QY of the dual-dye LSC was approximately 33% at 345 nm. The low QY of the RMC dye was improved significantly by adding a small amount of ZQD dye. Emissions of RMC were characterized as weak phosphorescence, with a relatively long emission lifetime of 5.05 μs. However, ZQD emitted strong fluorescence with a short fluorescence lifetime of 5.04 ns. The LSC embedded with both RMC and ZQD displayed a reduced lifetime of 4.22 ns at a ZQD emission wavelength of 490 nm and a slightly increased lifetime of 6.06 μs at the RMC emission wavelength of 670 nm. This indicates that energy was transferred from ZQD to RMC. A summary of optical properties of dye contained LSC plates are provided in Table S3 and Figure S9.

**Figure 5:**
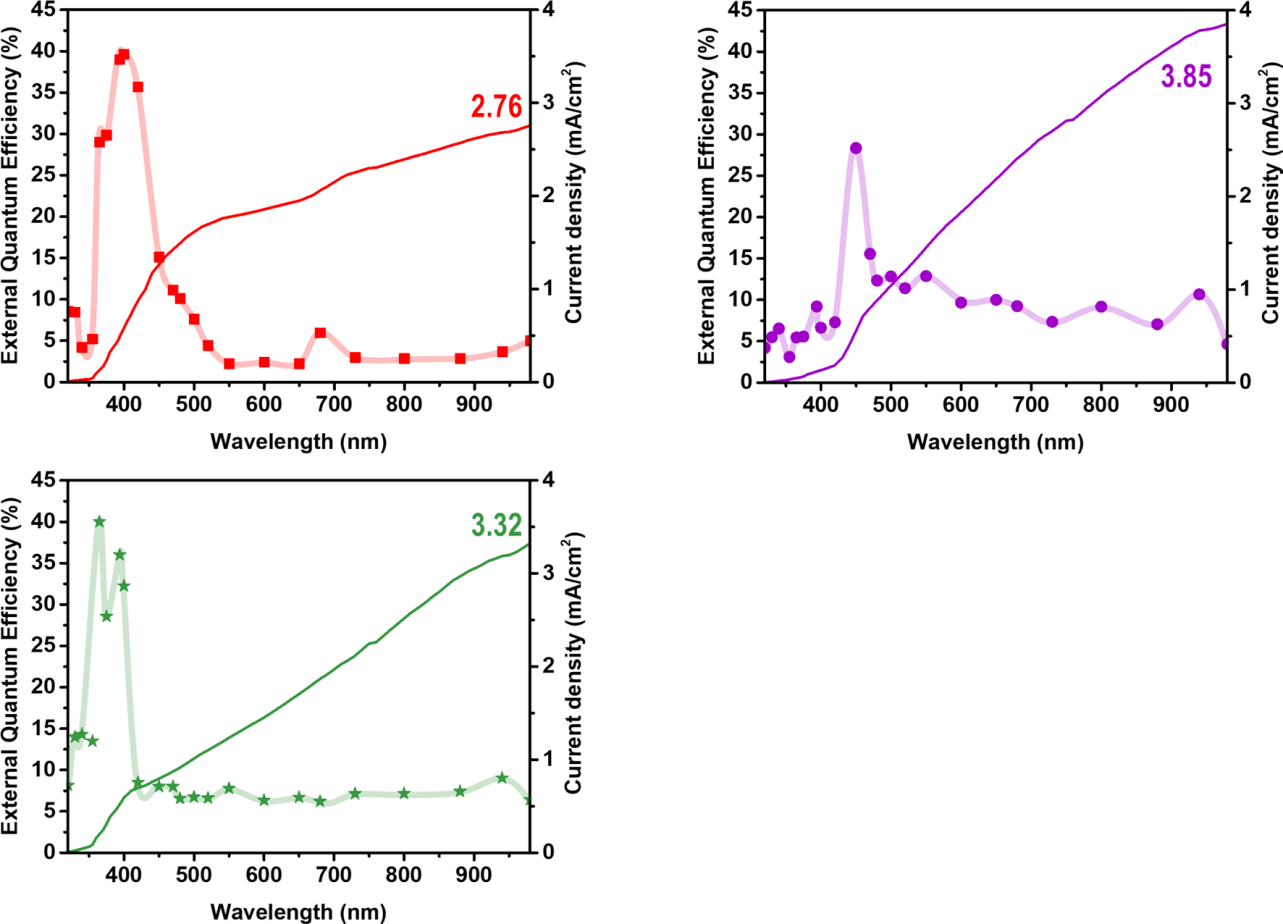
External quantum efficiency spectra of LSC systems with dye concentrations of C = 0.5 mg/mL for RMC (red) and ZQD (purple) and RMC-ZQD (green) with 0.5 and 0.08 mg/mL at a size of 2.5 cm × 2.5 cm × 3 cm as a function of wavelength.

We carried out external quantum efficiency (EQE) experiments to check the validity of the PCE and consistency of the *J*_*SC*_ obtained from J–V characteristics. The EQE is the current efficiency compared with that of incident photons. The integrated photocurrent density from the EQE spectra was compared with the current density extracted from J–V characteristics in PCE measurements. The EQE measurements were performed using a solar simulator with a 1000 W xenon lamp and a home-built apparatus consisting of 23 bandpass interference filters covering the 320–980 nm region^43^. The LSC had a single PV cell attached to one edge, with the other three edges painted black to block direct sunlight. The LSC device had a black background to prevent reflected light from entering from the rear of the device. In addition, light of other wavelengths was blocked so that only the light transmitted through the bandpass filter could reach the LSC. The EQE ($\lambda$) of the devices was calculated by Eq. (2)^44,45^:

2 $$ EQE(\lambda ) = EQE^{{ref}} (\lambda ) \times \frac{{I_{{LSC}} }}{{I_{{PV}}^{{ref}} }} $$

where $EQE^{{ref}} (\lambda )$ and $I_{{PV}}^{{ref}}$ are the EQE and short-circuit current of the silicon PV cell as a function of wavelength, respectively. *T*he short-circuit currents *I*_*LSC*_ of the LSC device were measured under a solar simulator as a function of wavelength. The spectrally resolved EQE (${{\lambda }}$) of the LSC system as a function of wavelength is shown in Fig. 5. Current densities of 2.76 ± 0.05 mA cm^−2^ in the RMC LSC, 3.85 ± 0.05 mA cm^−2^ in the ZQD LSC, and 3.32 ± 0.05 mA cm^−2^ in the dual-dye RMC-ZQD LSC were obtained by integrating the EQE spectra. The integrated currents from the EQE were consistent within 10% differences with the values of 3.04 ± 0.01 mA cm^−2^, 3.96 ± 0.05 mA cm^−2^, and 3.49 ± 0.05 mA cm^−2^ extracted from the J–V characteristics in Fig. 4.

**Figure 6:**
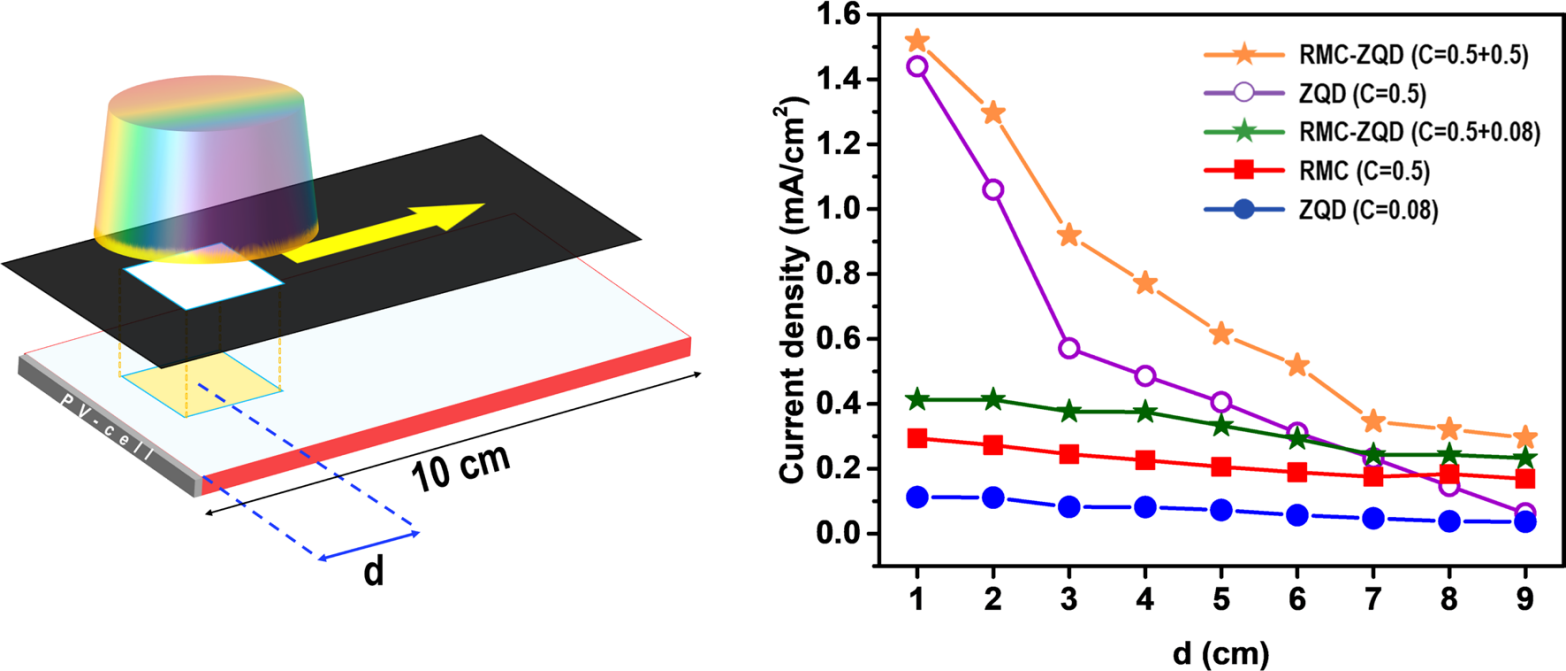
Schematic of the experiment for PV response of the LSCs as a function of distance between the PV cell and the excitation position. Current densities as a function of distance (d) were recorded on an excitation spot of area 2 cm × 2 cm.

To evaluate the reabsorption loss within the LSC waveguide, we compared the PV responses of the LSCs as a function of distance (*d*) between the PV cell and the excitation position (Fig. 6). The short-circuit current densities (*J*_*SC*_) of five LSCs with RMC, ZQD, and dual dyes (RMC-ZQD) were measured as a function of *d*. We fabricated a large LSC with dimensions of 7.5 cm ×  10.0 cm × 0.3 cm and attached a silicon PV cell to the edge (7.5 cm ×  0.3 cm). We used a mask so that a 2 cm × 2 cm area of LSC surface could be illuminated vertically at various positions from the Si PV cell. In the LSC containing 0.5 mg/mL of ZQD, as *d* increased, current density decreased exponentially, which is evidence of significant scattering and reabsorption losses. However, the LSC with RMC displayed a relatively constant *J*_*SC*_ as a function of *d*, indicating little reabsorption. In the dual-dye LSC with 0.5 mg/mL of RMC and 0.08 mg/mL of ZQD, current density increased due to the addition of ZQD, though it was constant as a function of *d*. When the concentration of ZQD was increased to 0.5 mg/mL in dual-dye system, rapid current decrease was observed as a function of *d*, but the reduction rate in *d* beyond several centimeters was not severe compared with that of the LSC with 0.5 mg/mL of ZQD alone indicating suppressed scattering effect by aggregated nano particles of ZQD. Although the PCE of an LSC containing 0.5 mg/mL of ZQD alone was as high as that of the dual-dye system, the exponential current–density decreased as a function of *d* make them unsuitable for practical application of large LSCs. The significant suppression of the reabsorption loss observed in the dual-dye LSC appeared to be due to uniform dispersion of RMC anions [Re_6_S_8_(NCS)_6_]^4−^ along with polar chalcogenide ZQD particles in the PMMA modified by (dMDAEMA)^+^ cations with hydrophobic also hydrophilic properties. This report suggests that chemically and optically favorable combinations of various dyes in a polymeric matrix can provide promising candidates for application of large-scale LSCs.

## Conclusions

We synthesized an inorganic–organic hybrid cluster (RMC) salt and inorganic QDs (ZQD) as luminophores and fabricated LSCs by encapsulating them within an amphiphilic PMMA matrix. The maximum PCE of 0.95% for the RMC alone embedded LSC was improved to 1.26% when small amounts of ZQD dye were added. High transparency and low reabsorption losses maintained due to uniform dispersion of two nano-sized chalcogenides dyes isolated in an amphiphilically modified polymer matrix by electrostatic and polar interactions. The extended harvesting coverage of solar spectrum by two dyes and synergetic energy transfer from ZQD to RMC resulted in a significantly enhanced PCE with reduced reabsorption and scattering, which was not possible with only RMC or ZQD alone. The combination of chemically and optically complementary dyes in the LSC will provide a new solution for development of a practical LSC system as transparent energy-harvesting windows for urban buildings.

## Experimental section

### Materials

To synthesize the hexarhenium cluster and core/shell QDs, 2-dimethyl amino ethyl methacrylate, 1-chlorododecane, cesium chloride (CsCl), potassium thiocyanate (KSCN), copper (Ι) iodide (CuI, 99.999%), gallium (III) iodide (GaI_3_, 99.99%), zinc chloride (ZnCl_2_, 98%), zinc stearate (Zn(St)_2_, 10–12% Zn basis), 1-dodecanethiol (DDT, 98%), oleylamine (OLA, 70%), oleic acid (OA, 90%), 1-octadecene (ODE, 90%), MMA, and azobisisobutylnitrile (AIBN) were purchased from Sigma Aldrich. Rhenium powder and sulfur powder (99.998%) were purchased from Alfa Aesar. All chemicals were used as received without further purification. For the photophysical measurements, acetonitrile (Duksan) was distilled over CaH_2_ in an argon atmosphere.

### Synthesis of the polymer-soluble cation (dMDAEMA)Cl

A 5 mL sample of 2-dimethyl amino ethyl methacrylate and 7.5 mL of 1-chlorododecane were dissolved in 50 mL of chloroform and heated to 50 °C for 18 h. After heating, the reacted solution was concentrated using a rotary evaporator and precipitated with cold diethyl ether. The precipitates were purified repeatedly with cold diethyl ether by centrifugation, filtered, and dried in a vacuum.

### Synthesis of the metal cluster compound (dMDAEMA)_4_[Re_6_S_8_(NCS)_6_] (RMC)

A 2 g of Re_6_S_8_Br_2_ and 3 g of KOH were placed in a carbon crucible and heated at 280 °C for 1 h. After heating, the cooled material was dropped slowly into a 7:3 mixture of ethanol and water using a funnel. The precipitate products, K_4_Re_6_S_8_(OH)_6_, were filtered, dried, and collected. 2 g of K_4_Re_6_S_8_(OH)_6_ in distilled water was heated and stirred at 80 °C for 1 h in air, and HCl was added dropwise to pH 1.5. After adding 1.16 g of CsCl, the solution was stirred for 2 h at 80 °C and cooled slowly to room temperature. To induce cation exchange, (dMDAEMA)Cl was dissolved in 50 mL of distilled water and then added dropwise into the Cs_4_Re_6_S_8_Cl_6_ solution. The solution was filtered through a funnel and dried at room temperature to obtain (dMDAEMA)_4_[Re_6_S_8_Cl_6_]. A solid mixture of 0.200 g (0.078 mmol) of (dMDAEMA)_4_[Re_6_S_8_Cl_6_] and 5.0 g (51.58 mmol) of KSCN was heated at 200 °C for 1 h to obtain a suspension in liquid KSCN. After the sample was cooled to room temperature, 100 mL of water was added to the brown solid, and the mixture was stirred for 20 min. The yellow solid of (dMDAEMA)_4_[Re_6_S_8_(NCS)_6_] was collected by filtration and re-dissolved in acetonitrile to remove the insoluble solid^36^.

### Synthesis of core/shell quantum dots (ZQDs)

For typical synthesis of ternary copper-deficient CuGaS (CGS) as core QDs, Cu/Ga precursor was mixed with 0.0625 mmol of CuI, 0.5 mmol of GaI_3_, and 1 mmol of sulfur in a three-neck flask with 1.5 mL of 1-dodecanethiol (DDT) and 5 mL of oleylamine (OLA). The mixture was degassed by heating to 120 °C and further heated to 240 °C under an argon gas flow. The reaction was maintained at 240 °C for 5 min to allow growth of core QDs. A series of Zn-doped CuGaS QDs, Zn_x_Cu_1-x_GaS, was synthesized by adding 1.5 mmol of ZnCl_2_. Quaternary core Zn_x_Cu_1−x_GaS QDs were subjected to the following multiple-shelling forming reaction. The first ZnS stock solution, prepared by dissolving 8 mmol of reagent-grade zinc acetate in 8 mL of OA and 4 mL of ODE, was introduced slowly into the core QD solution at 240 °C and allowed to react for 75 min. Subsequently, the second ZnS stock solution, consisting of 4 mmol of Zn acetate, 4 mL of OA, 2 mL of DDT, and 2 mL of ODE, was injected slowly, followed by 30 min of reaction at 240 °C. Next, another ZnS solution containing 4 mmol of Zn stearate (10–12% zinc basis), 4 mL of ODE, and 2 mL of DDT was injected, and this final shelling reaction was allowed to proceed at 250 °C for 1 h. The purification was repeated with a conventional work-up process in which unreacted QDs were precipitated with excess ethanol and centrifuged at 9000 rpm for 10 min in a solvent of hexane/ethanol^42^.

### Synthesis of hybrid LSC materials

For the LSC with an embedded rhenium cluster dye, RMC was dissolved in MMA solution at concentrations of 0.25, 0.5, 0.75, 1.0, and 1.25 mg/mL in glass vials and sonicated for 90 min in an ultrasound bath to disperse them thoroughly^46^. After sonication, an initiator, AIBN, was added at an MMA:AIBN ratio of 1 mL:6 µL. This mixture was sonicated for 2 h to produce a homogeneous solution and kept in a dry oven for 24 h at 65 °C in a 70 mL round glass vial mold. The final solid-state products were transparent and tintless. The highest PCE obtained from the fabricated device involved a concentration of 0.5 mg/mL of RMC. The LSC with dual dyes was prepared by adding 0.04, 0.08, 0.16, 0.33, and 0.5 mg/mL of ZQD QDs dissolved in THF solution to RMC dissolved in MMA solution with a concentration of 0.5 mg/mL.

### Module fabrication

To produce LSC devices, final solid-state plates were cut into square 2.5 cm × 2.5 cm plates and 7.5 cm × 10.0 cm with a thickness of 3 mm. For QY measurement, we cut plates to a size of 1 cm × 1 cm × 0.3 cm. To ensure a clear appearance, the plates were polished using sandpaper in the order of size (mm): 100, 600, 1000, 2000 and polished using cloth with MD-Nap 200 mm diameter, purchased from Struers. At the edge of the LSC plate, silicon PV cells (Narec Solar) with an efficiency of 16 ± 0.19% at 1 sun of illumination were attached using MMA solution to ensure the same refractive index as the PMMA matrix.

### Characterization

For absorption and emission measurements, solution spectra were recorded in the range of 280–800 nm on an Agilent YoungIn G1103A UV/Vis spectrophotometer. Photoluminescent spectra were recorded in the range of 450–900 nm on a Photon Technology International, Quanta Master 400 scanning spectrofluorometer. Measurements were carried out at 298 K. The THF solutions were de-aerated using bubbling argon gas prior to measurements. A quartz cell (Hellma, beam path length = 1.0 cm) was employed for solution samples. The absorption and transmittance spectra of solid samples were recorded in a range of 280–800 nm on a Cary 5000 at 298 K. The excitation and emission spectra of solid hybrid samples were measured on a Perkin Elmer LS 55 fluorescence spectrometer equipped with a xenon lamp at 298 K. The excitation spectra were recorded in a range of 250–600 nm at the maximum emission wavelengths of 550 and 670 nm. The emission spectra were recorded in a range of 500–900 nm with an excitation wavelength of 345 nm. The TEM measurement was performed using the HRTEM (JEM-2100F) at the National Research Facilities and Equipment Center (NanoBioEnergy Materials Center) supported by Korea Basic Science Institute (National Research Facilities and Equipment Center) (2020R 1A 6C 101B194) at Ewha Womans University.

The EQE and PCE of LSC devices were characterized by illumination under a solar simulator (Mc Science K201 LAB50, Oriel). For EQE measurements, silicon PV cells attached at the edge of the LSC were masked with black electrical tape to avoid direct illumination by sunlight and ensure the actual current was generated from emission of the LSC. The EQE spectrum of the attached cell was obtained in a range of 300–1100 nm on a K3100 EQX (McScience, Korea) apparatus. The lifetimes of dyes were measured with a Fluorolog-3 time-correlated single-photon counting system, and the data were fit to bi-exponential decay functions. The *Isc* of the device as a function of irradiation at various wavelengths was measured under a solar simulator using 23 bandpass interference filters. The absolute QY of the RMC-ZQD-polymer hybrid was measured by a UV–near infrared absolute PLQY measurement system (Hamamatsu Photonics, C11347-11), which comprised a xenon excitation light as a source and an integrated sphere, with a measurement excitation wavelength fixed at 345 nm. The size of RMC-ZQD-polymer hybrid was 1 cm × 1 cm × 0.3 cm.

## Supplementary Information

Supplementary Information.
