# Supplementary material for: Quantum dot assisted luminescent hexarhenium cluster dye for a transparent luminescent solar concentrator
Source: Sci Rep. 2021 Jul 5;11:13833. doi: 10.1038/s41598-021-93223-7 (PMC8257676; doi:10.1038/s41598-021-93223-7)
Supplement: Supplementary file 1 — Supplementary Information. [file 41598_2021_93223_MOESM1_ESM.docx]

**Supplementary material**

**Quantum Dot Assisted Luminescent Hexarhenium Cluster Dye for a Transparent Luminescent Solar Concentrator**

Jun Choi, Kyungkon Kim, Sung-Jin Kim*

Department of Chemistry and Nano Science, Ewha Womans University,

Seoul, 120-750, Korea

*Email: sjkim@ewha.ac.kr


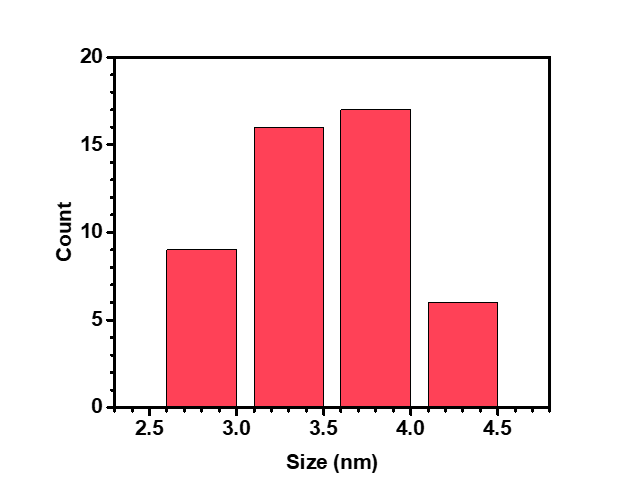


**Figure S1.** (a) A histogram of ZnCuGaS/ZnS core/shell quantum dot (ZQD) sizes with a mean diameter of 3.62 nm.

**
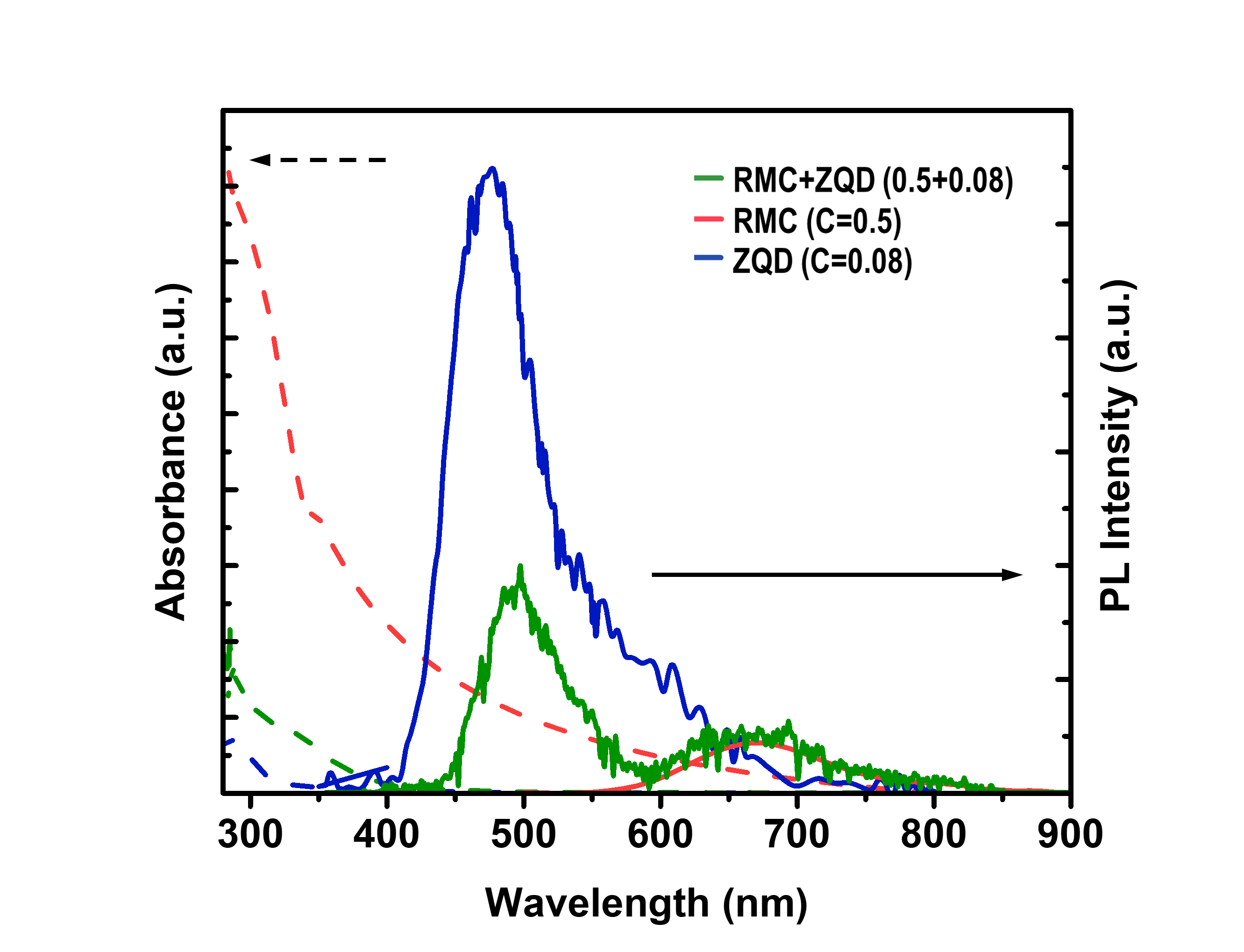
**

**Figure S2.** Absorbance (dashed line) and PL (solid line) spectra of RMC + ZQD LSC in the range of 280 to 900 nm (excitation wavelength of 345 nm). The emission peak of ZQD in the mixture appeared at 490 nm, which was slightly red-shifted from that of ZQD alone at 471 nm.

**
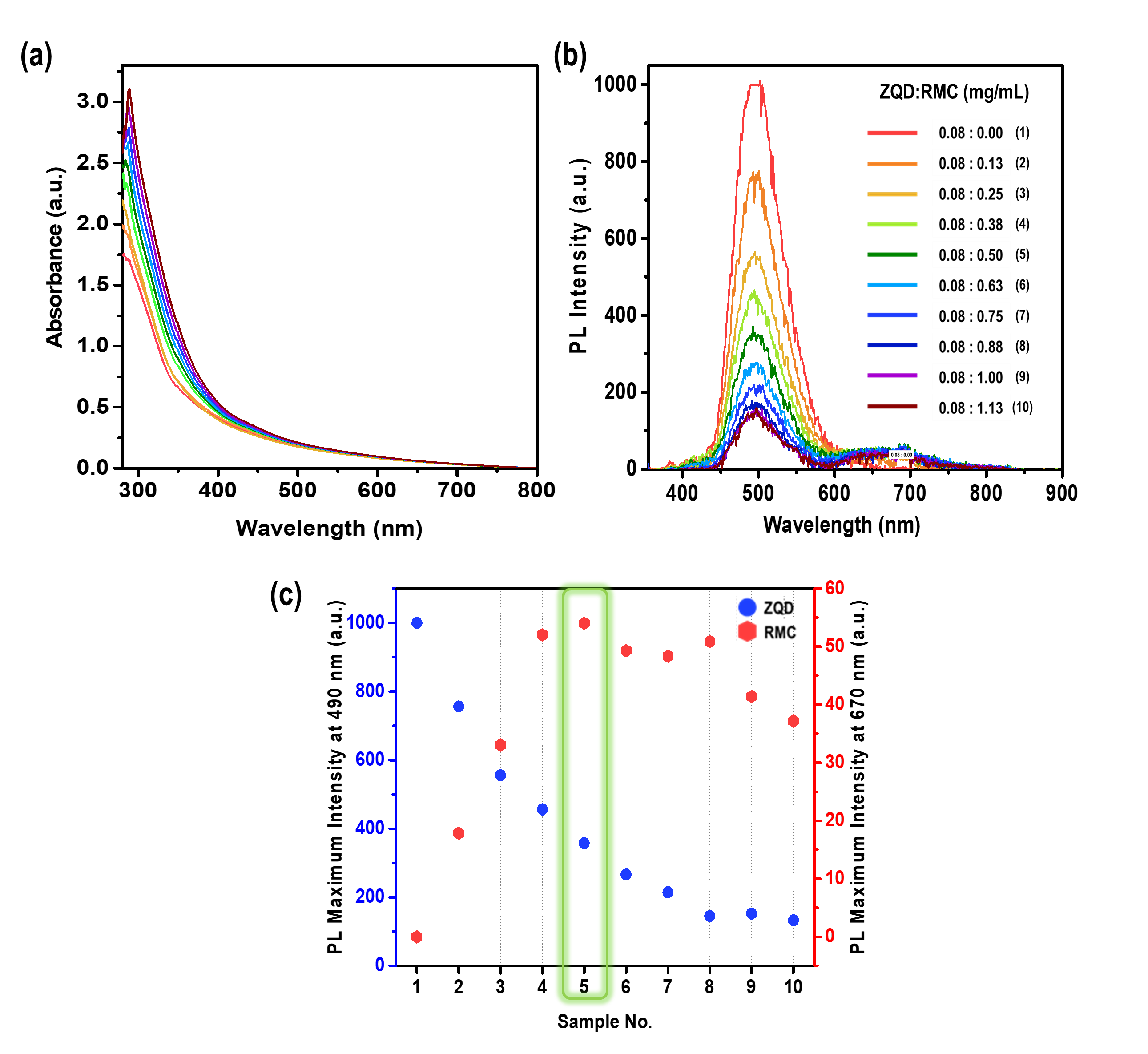
**

**Figure S3.** UV-vis absorption (a) and photoluminescence (b) (λex = 345 nm) spectra for dual-dye solution in argon-saturated THF with 0.08 mg/mL ZQD and increased concentration (0–1.13 mg/mL) of RMC (sample No. 1-10). (c) Photoluminescence intensities of ZQD (blue) at 490 nm and RMC (red) at 670 nm. As the concentration of RMC increased, the PL intensity of ZQD at 471 nm decreased and red-shifted slightly to 490 nm, indicating that energy was transferred from ZQD to RMC. The PL intensity of RMC at 670 nm peaked when 0.5 mg/mL RMC was added to 0.08 mg/mL ZQD (sample No. 5) and remained high until 0.88 mg/mL RMC.

| Sample No. | ZQD:RMC  (mg/mL) | Donor quenching  efficiency | Acceptor sensitization efficiency | |  |
| --- | --- | --- | --- | --- | --- |
|  |  | **(1-F_D_/F_0_)** | | **Φ_D_ (F_A_-**$\mathbf{F}_{\boldsymbol{A}}^{\mathbf{0}}$**)/(Φ_A_** $\mathbf{F}_{\boldsymbol{D}}^{\mathbf{0}}$**)** | |
| 1 | 0.08:0.00 | 0.00 | | 0.00 | |
| 2 | 0.08:0.13 | 0.24 | | 0.07 | |
| 3 | 0.08:0.25 | 0.44 | | 0.14 | |
| 4 | 0.08:0.38 | 0.54 | | 0.21 | |
| 5 | 0.08:0.50 | 0.64 | | 0.22 | |
| 6 | 0.08:0.63 | 0.73 | | 0.20 | |
| 7 | 0.08:0.75 | 0.79 | | 0.20 | |
| 8 | 0.08:0.88 | 0.85 | | 0.21 | |
| 9 | 0.08:1.00 | 0.85 | | 0.17 | |
| 10 | 0.08:1.13 | 0.87 | | 0.15 | |

**Table S1**. Summary of donor quenching and acceptor sensitization. ^1^ Where **F_D_** is PL intensity of the donor (ZQD), and $\mathbf{F}_{\boldsymbol{D}}^{\mathbf{0}}$ is PL intensity of sample No. 1 with 0.08 mg/mL ZQD alone without acceptor. **Φ_D_** (0.78), and **Φ_A_** (0.19) are the quantum yield of donor and acceptor, respectively. **F_A_** represents the PL intensity of the acceptor (RMC) at 670 nm, and $\mathbf{F}_{\boldsymbol{A}}^{\mathbf{0}}$ represents the PL intensity of sample No. 1 at 670 nm without RMC.


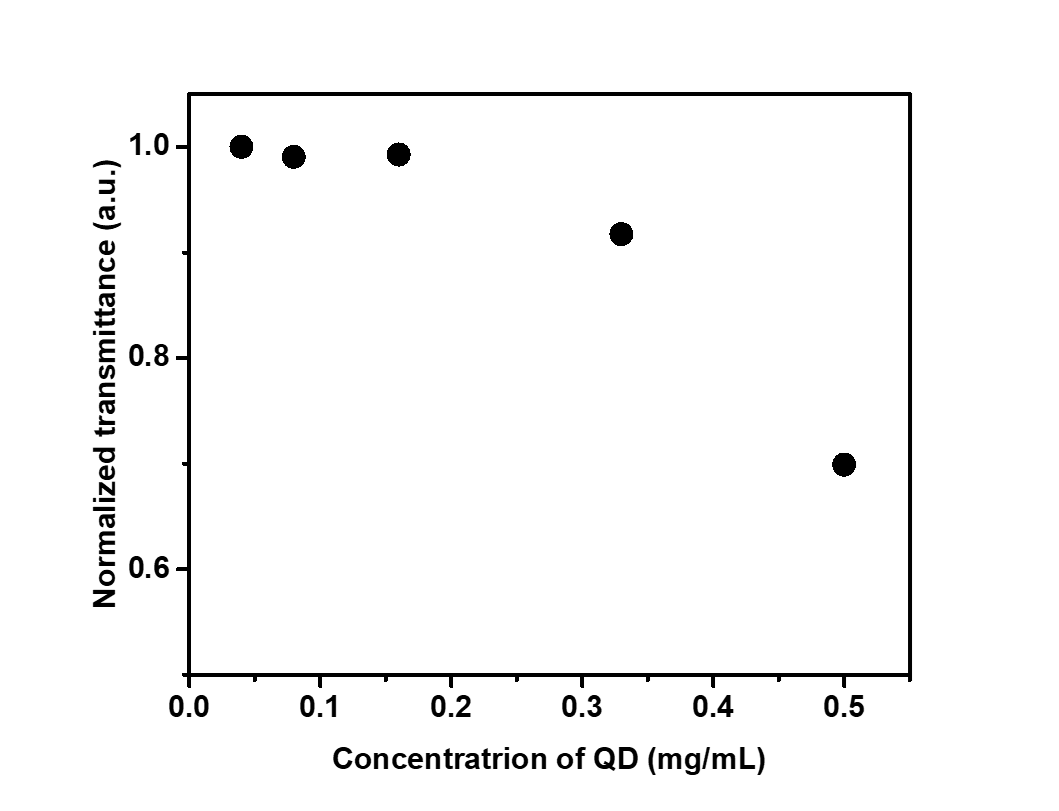


**Figure S4.** Transmittance of LSC with 0.04, 0.08, 0.16, 0.33, and 0.5 mg/mL of ZQD. When the ZQD concentration is higher than 0.33 mg/mL, the transparency decreases due to the scattering of light by aggregated QDs.

|  | x | y | CRI |
| --- | --- | --- | --- |
| PMMA | 0.3335 | 0.3336 | 97 |
| RMC (C = 0.5) | 0.3453 | 0.3500 | 99 |
| RMC-ZQD  (C = 0.5 + 0.08) | 0.3426 | 0.3461 | 98 |
| ZQD (C = 0.08) | 0.3343 | 0.3351 | 97 |
| ZQD (C = 0.5) | 0.4206 | 0.4032 | 99 |

**Table S2**. Color coordinates of the prepared LSCs calculated using the CIE 1931 chromaticity diagram.

| Concentration (mg/mL) | J_SC_(mA cm^-2^) | V_OC_ (V) | FF | PCE (%) |
| --- | --- | --- | --- | --- |
| C = 0.25 | 2.355 | 0.513 | 0.685 | 0.83 |
| C = 0.50 | 2.892 | 0.514 | 0.642 | 0.95 |
| C = 0.75 | 2.327 | 0.508 | 0.723 | 0.85 |
| C = 1.00 | 2.490 | 0.499 | 0.654 | 0.81 |
| C = 1.25 | 2.326 | 0.508 | 0.674 | 0.80 |


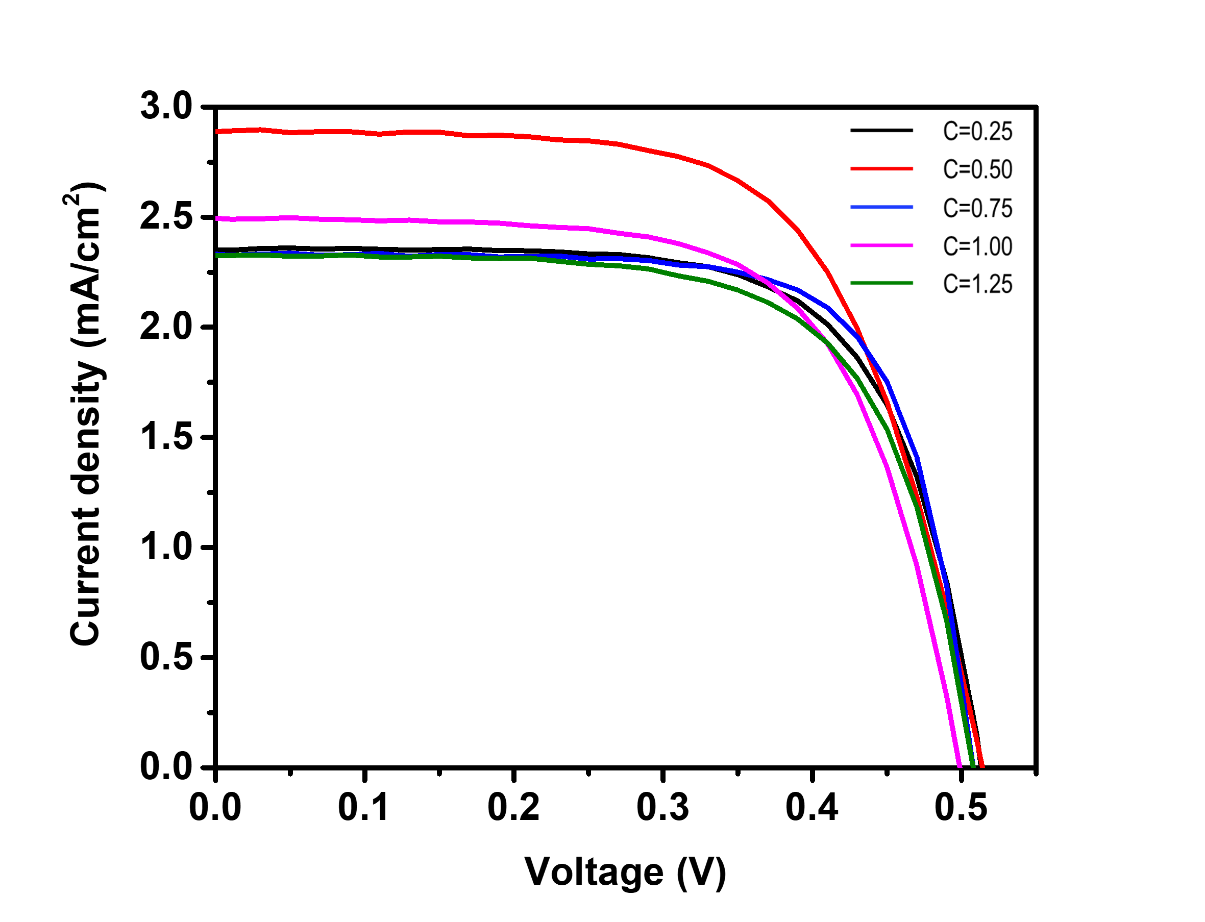


**Figure S5.** J-V curves of the RMC LSC panel at various concentrations of C = 0.25, 0.50, 0.75, 1.0 and 1.25 mg/mL.

| Concentrations (mg/mL) | J_SC_(mA cm^-2^) | V_OC_ (V) | FF | PCE (%) |
| --- | --- | --- | --- | --- |
| C = 0.04 | 0.918 | 0.42 | 0.694 | 0.27 |
| C = 0.08 | 1.452 | 0.405 | 0.491 | 0.29 |
| C = 0.16 | 1.557 | 0.507 | 0.677 | 0.53 |
| C = 0.32 | 3.325 | 0.527 | 0.592 | 1.04 |
| C = 0.50 | 3.459 | 0.535 | 0.675 | 1.25 |


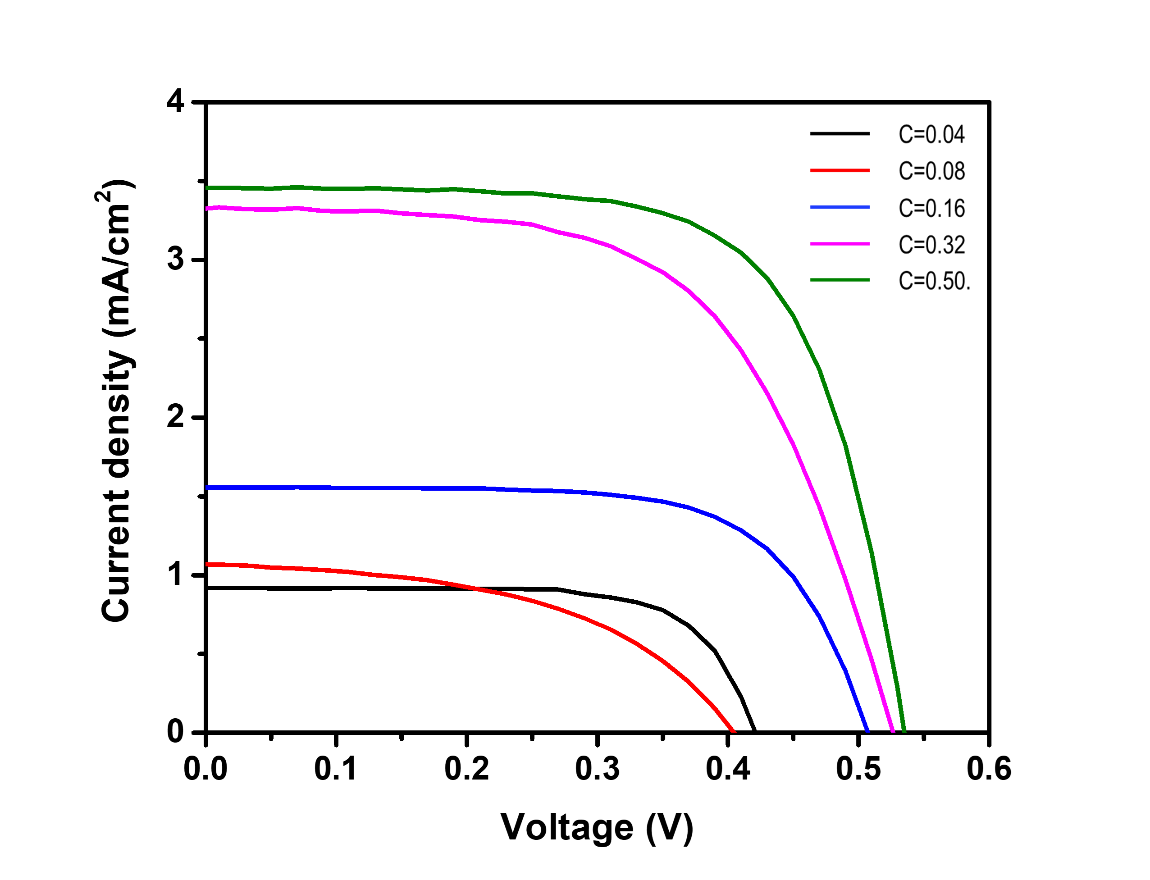


**Figure S6.** J-V curves of the ZQD LSC panel at various concentrations of 0.04, 0.08, 0.16, 0.33, and 0.5 mg/mL.


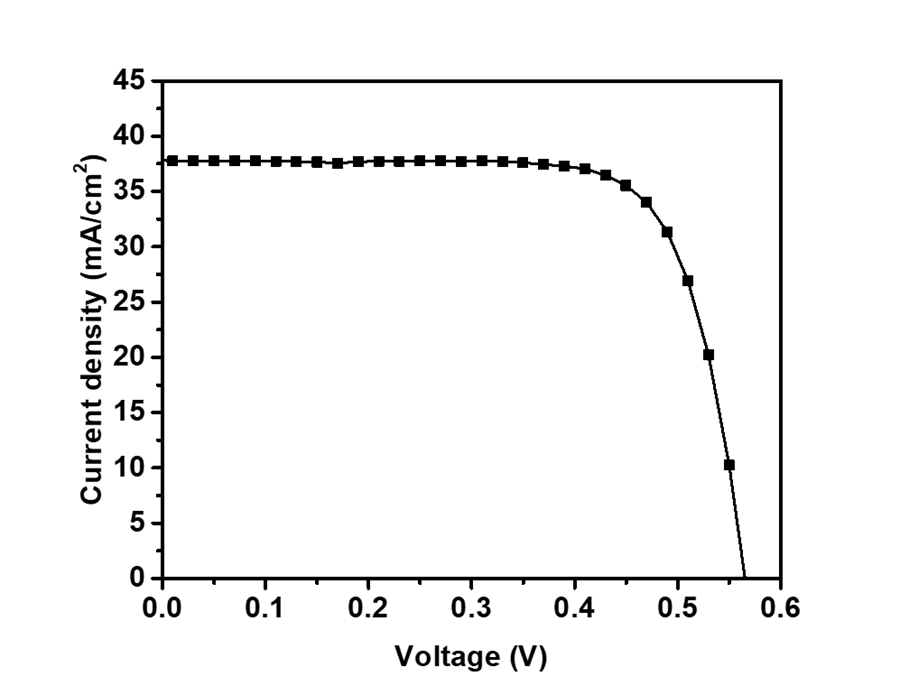


|  | I_SC_(mA) | V_OC_ (V) | FF | PCE (%) | Average  PCE (%) |
| --- | --- | --- | --- | --- | --- |
| #1 | 37.823 | 0.565 | 0.748 | 15.985 | 16 ± 0.19 |
| #2 | 37.920 | 0.563 | 0.739 | 15.777 |  |
| #3 | 37.702 | 0.567 | 0.756 | 16.161 |  |

**Figure S7.** J-V characterizations of silicon PV cells, with an average PCE of 16 ± 0.19% (1 sun, AM 1.5 G).


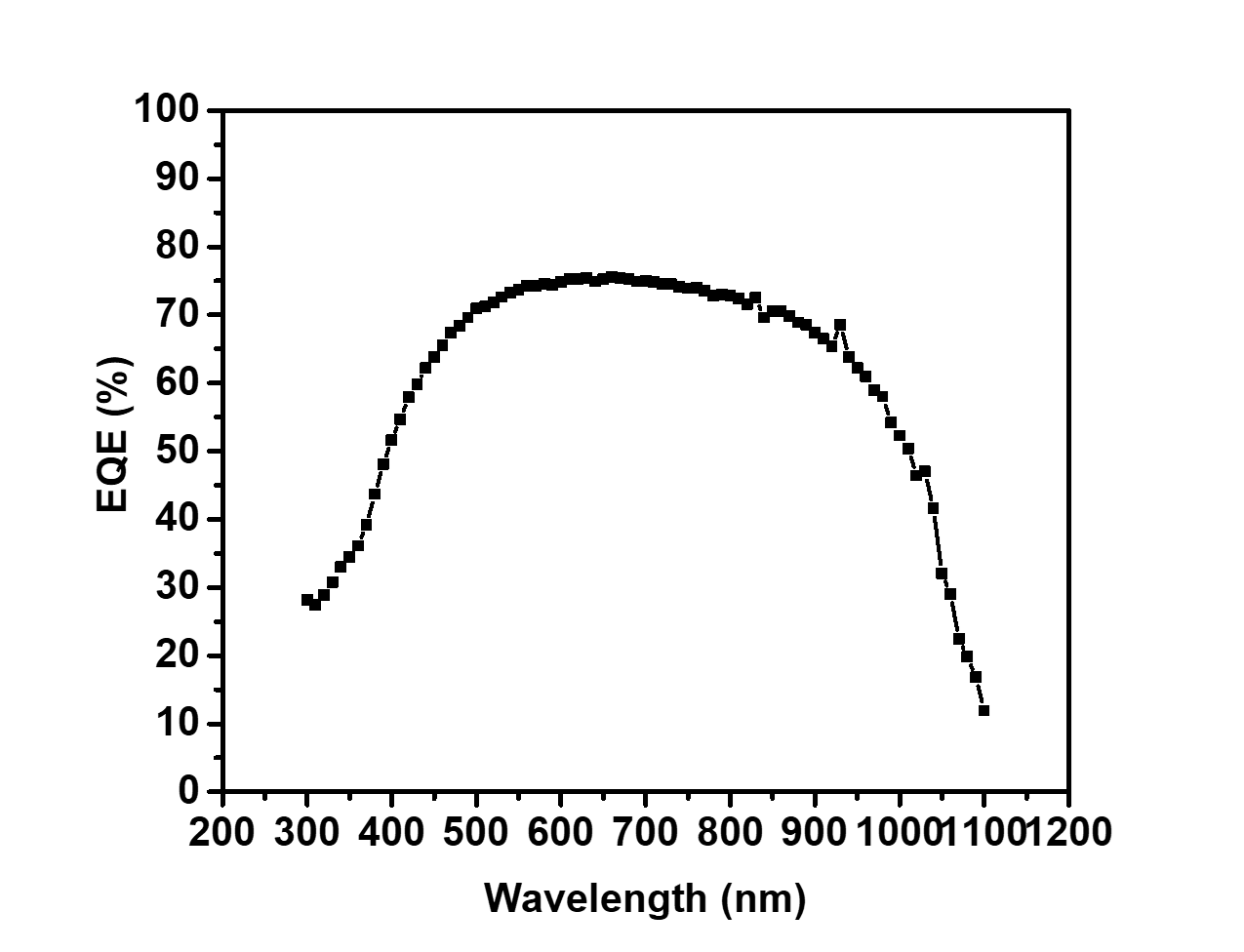


**Figure S8.** EQE spectra of the silicon PV cells.

**Table S3.** Optical properties of LSC plates embedded with single dyes of **RMC** and **ZQD**, and with the dual-dye **RMC-ZQD**. $\phi$ = quantum yield, Em(max) = maximum emission wavelength, ɛ = extinction coefficient, τ = amplitude weighted average lifetime.

|  | $\phi$ | Em(max) | $\varepsilon$ (M^-1^ cm^-1^) | $\tau$ (s) |
| --- | --- | --- | --- | --- |
| RMC | 0.19 | 670 | 8.41 ×10^4^ | 5.05 × 10^-6^ |
| ZQD | 0.78 | 471 | 7.72 × 10^3^ | 5.04 × 10^-9^ |
| RMC-ZQD | 0.33 | 490, 670 | 2.23 × 10^4^ | 6.06 ×10^-6^,  4.22×10^-9^ |

**
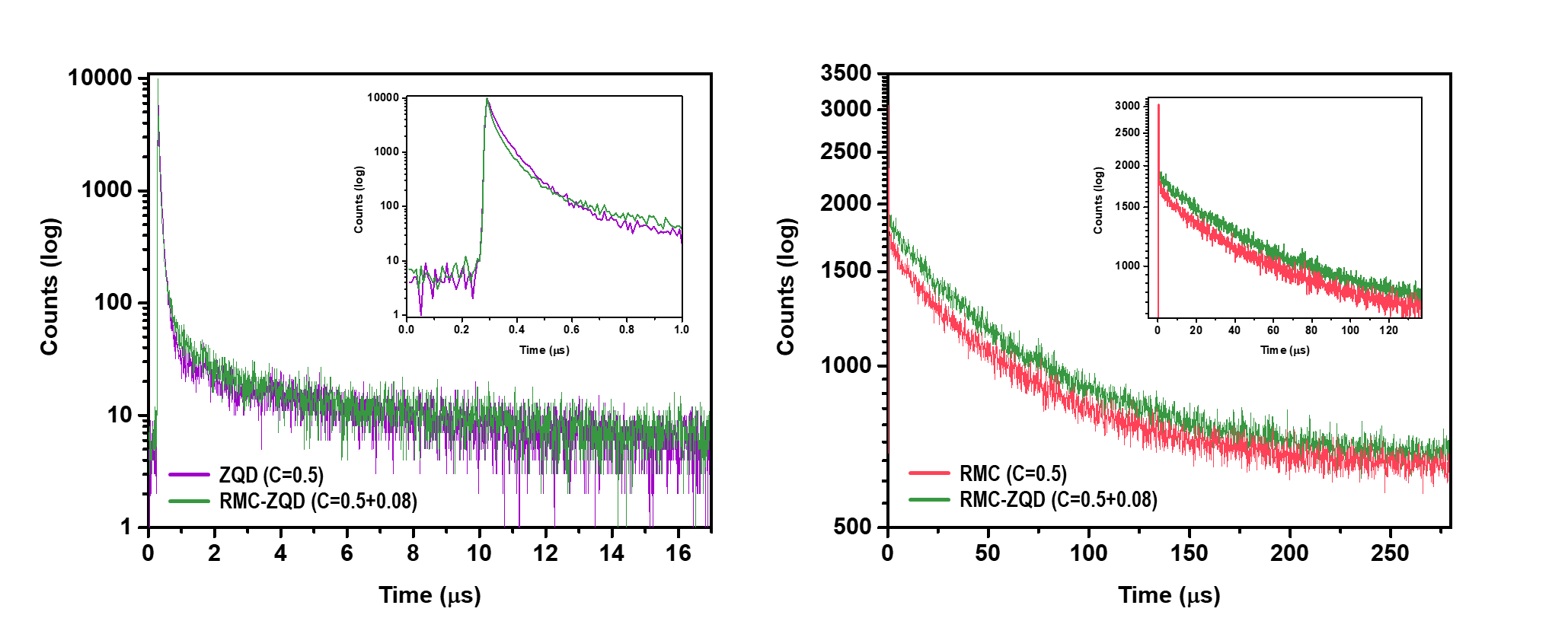
Figure S9.** Time-resolved PL intensity of LSCs with ZQD, RMC, and RMC-ZQD ($\lambda$_ex_ = 374 nm, $\lambda$_em_ = 490 nm [left],$\lambda$_em_ = 670 nm [right]).

**References**

1 Oh, E. *et al.* Energy Transfer Sensitization of Luminescent Gold Nanoclusters: More than Just the Classical Forster Mechanism. *Sci Rep-Uk* **6**, 35538, (2016).
